# Portuguese Physical Literacy Assessment - Observation (PPLA-O) for adolescents (15–18 years) from grades 10–12: Development and initial validation through item response theory

**DOI:** 10.3389/fspor.2022.1033648

**Published:** 2022-12-15

**Authors:** João Mota, João Martins, Marcos Onofre

**Affiliations:** ^1^ Centro de Estudos em Educação, Faculdade de Motricidade Humana, Cruz-Quebrada-Dafundo, Oeiras, Portugal; ^2^ UIDEF, Instituto de Educação, Lisbon, Portugal; ^3^ School of Education, Sports Studies and Physical Education Programme, University College Cork, Cork, Ireland

**Keywords:** physical literacy, assessment, physical education, development, construct validity, reliability, high school, adolescence

## Abstract

**Introduction:**

Aims of these studies were to develop the Portuguese Physical Literacy Assessment Observation instrument (PPLA-O) to assess the physical and part of the cognitive domain of Physical Literacy (PL) through data collected routinely by Physical Education (PE) teachers; and to assess the construct validity (dimensionality, measurement invariance, and convergent and discriminant validity) and score reliability of one of its modules [Movement Competence, Rules, and Tactics (MCRT)].

**Methods:**

Content analysis of the Portuguese PE syllabus and literature review were used for PPLA-O domain identification. Multidimensional Item Response Theory (MIRT) models were used to assess construct validity and reliability, along with bivariate correlations in a sample of 515 Portuguese grade 10–12 students (*M*_age_ = 16, SD = 1).

**Results:**

PPLA-O development resulted in an instrument with two modules: MCRT (22 physical activities) and Health-Related Fitness (HRF; 5 protocols); both assessed with teacher-reported data entered in a spreadsheet. A two correlated dimensions Graded Response Model (Manipulative-based Activities [MA], and Stability-based Activities [SA]) showed best fit to the MCRT data, suggesting measurement invariance across sexes, and adequate to good score reliabilities (MA = .89, and SA = .73). There was a moderate to high correlation (*r* = .68) between dimensions, and boys had higher scores in both dimensions. Correlations among MCRT scores and HRF variables were similar in magnitude to previous reports in meta-analysis and systematic reviews.

**Conclusions:**

PPLA-O is composed of two modules that integrate observational data collected by PE teachers into a common frame of criterion-referenced PL assessment. The HRF module uses data collected through widely validated FITescola® assessment protocols. The MCRT makes use of teacher-reported data collected in a wide range of activities and movement pursuits to measure movement competence and inherent cognitive skills (*Tactics* and *Rules*). We also gathered initial evidence supporting construct validity and score reliability of the MCRT module. This highly feasible instrument can provide Portuguese grade 10–12 (15–18 years) PE students with feedback on their PL journey, along with the other instrument of PPLA (PPLA-Questionnaire). Further studies should assess inter and intra-rater reliability and criterion-related validity of its two modules.

## Introduction

Physical literacy (PL) is a holistic concept composed of four interrelated domains: physical, emotional/psychological, cognitive, and social. It comprises skills and attributes that individuals show through physical activity (PA) and movement throughout their lives (1, 2). This concept is also at the heart of quality Physical Education (PE) for school-aged children and adolescents (3, 4).

Two crucial elements within the physical domain of PL are movement competence (MC) and health-related fitness (HRF), as they are conceptualized as part of a spiral of engagement that leads to increased PA participation in children, which might strengthen into adolescence (5, 6)—a stage in life in which we will focus, given their concerning low levels of PA (7). However, if the goal is *meaningful* and involved PA participation, its decision-making and tactical aspects (elements of the cognitive domain of PL) need to be also considered (2, 8–10).

Development of MC, HRF, and decision-making is an explicit or implicit part of some PE syllabi (11), as is the case of Portugal (12–15), where data on MC—through an authentic assessment lens, that integrates movement and decision-making skills (16)—and HRF of students is routinely collected by PE teachers. These teachers are qualified movement professionals that observe students in various settings (17, 18), and may be in a privileged position to assess multiple aspects of student development (19, 20). While HRF assessment makes use of standardized protocols (FITescola®; 21) that produce generalizable and interpretable data for educational and research stakeholders, within and outside of schools, this has not been the case for the assessment of MC.

One option to solve this issue would be the use of MC assessment batteries; however, these suffer from multiple drawbacks: (1) they require additional training and/or lesson time for correct application (22), and so lower their feasibility in PE settings; (2) they focus mostly on children (23); (3) those available for adolescents are generally product-oriented (24), providing assessment only in discrete, low-generalization tasks (25) that lack the needed ecological validity (6) to understand engagement in advanced physical experiences in a variety of domains and environmental constraints (25, 26)—a characteristic that defines motor development in adolescence (27, 28); and, (4) they neglect the decision-making aspects previously mentioned, requiring separate use of other instruments, that are however, limited to formalized games (29, 30).

This issue motivated the development of a criterion-referenced instrument that could frame observational data collected by teachers in the physical and cognitive domains into the Portuguese Physical Literacy Assessment (PPLA) tool, which already counts with measures to assess all other domains of PL in adolescents (aged 15–18) (31–33).

Our aims for the following studies were to (a) develop the PPLA-Observation (PPLA-O) based on the review of relevant conceptual frameworks and the Portuguese PE syllabus—resulting in two modules, the Movement Competence, Rules, and Tactics (MCRT) module and the Health-Related Fitness (HRF) module; (b) investigate the dimensionality structure of MCRT module through Item Response Theory (IRT) methods; (c) test this structure for differential item functioning (DIF) according to sex, as comparisons between sexes are likely in the future, due to suggested differences in object-controlling/manipulative skills (34); (d) establish support for convergent and discriminant validity, and score reliability for this module. A secondary aim was to draw inferences for scoring and criterion-referenced cut-scores mechanisms. We did not focus on validation of the HRF module as it comprises measures (i.e., FITescola® protocols) that have already published evidence to support validity and reliability—further details in the Results section.

## Materials and methods

### Overview

The development and testing of the PPLA-O followed a common philosophy—centered in providing a criterion-referenced and feasible tool for PE use—and multiple-phase methodology to that of the other part of PPLA: PPLA-Questionnaire (PPLA-Q; 31). It was inspired by the physical and cognitive domains of the PL model proposed in the APLF (2, 35), and by the Portuguese PE syllabus (12–15).

These studies entailed domain identification and measure selection, resulting in an instrument with two modules: HRF and MCRT; followed by content analysis of the PPES according to chosen taxonomies to ensure content validity. A pilot test evaluated feasibility of data entry for PE teachers. Finally, we assessed the dimensionality and reliability of the *Movement Competency, Rules, and Tactics* module. Since the HRF module is grounded in widely used and reported protocols (i.e., FITescola©; 21), no validation was done. In all phases, adherence to standards for instrument development and validation was sought (36, 37).

### Domain identification and measure selection

Similar to the procedures conducted for the development of the PPLA-Q (31), a theoretical framework was established for each of the nine selected elements in the physical and cognitive domains based on a literature review of relevant theories in the fields of motor development, physical fitness, and PE; supported by previous review efforts by the APLF team (35), and analysis of the Portuguese PE syllabus (PPES; 12–14). Afterward, each selected element was mapped into the two-level PPLA framework (31). This framework establishes a *Foundation* (initial development that enables participation in movement and PA) and *Mastery* level (relational understanding and application of skills) of development for each element, based on the original APLF work, and the structure of observed learning outcomes taxonomy (SOLO; 38). Operational definitions per element and level were based on the APLF (2). Then, based on the PPES and its assessment norms, measures, or instruments for each element were selected to maximize feasibility and ecological validity.

Since, as we will detail in the Results section, the PPES uses an integrated criterion-referenced assessment of movement competencies, along with rules' knowledge and tactical development, a summative content analysis of the syllabus was conducted (39) to study possible factorial structures that would allow disentangling these various elements from each other. Coding was made by the lead investigator, using a deductive categorization (40) with categories extracted from the respective theories or models; as no specific taxonomy existed for the *Rules* element, an inductive approach was taken. For the *Movement Competence* skills, sport/specialized skills in each chosen activity were assessed for the diversity of movement skills required in its execution, based on Gallahue's (27) taxonomy of Locomotion, Manipulative, and Stability movement skills, along with Dudley's (9) taxonomy for Moving with equipment (or Object Locomotion). For the *Tactics* element, the diversity of tactical actions was counted according to the Game Performance Assessment System (30).

### Pilot testing

Concurrent with the pilot test of the PPLA-Q (31) in November 2020, two PE teachers from the involved classes were asked to complete the resulting PPLA-O from the previous phase. PPLA-O took the form of a spreadsheet file (Supplementary Material S1) where teachers could enter all results from the selected (1) proficiency levels for MCRT—ordinal code, and (2) HRF protocols—continuously coded, except for Shoulder Stretch, which was coded as a binary variable; along with demographic information for each student. Feasibility was assessed through qualitative comments on the clarity of the provided instructions for data insertion, and identification of *bugs* in the automated spreadsheet files used to generate unique codes for each student (to assure anonymity) and insert data.

### IRT analysis of the movement competence, rules, and tactics module

#### Participants

This study used the same sample as previous PPLA-Q validation studies. Sampling procedures are fully described in previous work (32). Briefly, a convenience sample of 521 grade 10–12 students from 25 classes in 6 public schools in Lisbon metropolitan area was used. Recruitment was stratified by grade and course major according to population percentage quotas. Schools from diverse socioeconomic backgrounds were chosen to increase sample representativeness. Student sample characteristics are summed up in Table 1. Data about students was reported by 22 PE teachers. The sample size conformed to recommendations for multidimensional graded response models (GRM) (41).

**Table 1:** Student sample characteristics.

Characteristic	*N* = 521^a^
**Sex (*n* miss. = 2)**	
Female	303 (58%)
Male	216 (42%)
Age	16 (1)
**Grade**	
10	208 (40%)
11	144 (28%)
12	169 (32%)
**Major**	
Economics	76 (15%)
Humanities	166 (32%)
STEM	279 (54%)
**School**	
School 1	40 (8%)
School 2	67 (13%)
School 3	21 (4%)
School 4	71 (14%)
School 5	208 (40%)
School 6	114 (22%)

STEM, sciences, technology, engineering, and math.

^a^ Statistic presented: *n* (%); *M* (SD).

#### Measures and procedures

PPLA-O was completed by the PE teachers (*N* = 22) of each class from January to March 2021. Data collection for this tool was concurrent with the one for PPLA-Q validation studies (32, 33). Upon acceptance to participate, teachers were sent the PPLA-O matrix and were asked to return the latter upon data collection of the PPLA-Q. Since a lockdown was in effect due to the COVID-19 pandemic for most of the data collection, teachers were asked to provide the most recent data before lockdown, according to the levels provided in the PPES and protocols of the FITescola®. Despite not being part of the PPLA-O, height and weight information were collected to calculate body mass index (BMI) for each student. This measure would be used for testing relevant correlations with measures in the MCRT module.

#### Analysis

All analyses were performed in RStudio (42) with R 4.1.0 (43). Partial PE proficiency levels (e.g., partial Elementary level) were collapsed into the adjacent lower category to equalize assessment across schools—since it is common for each school to define their criteria for these partial levels to motivate students.

Descriptive statistics were generated using the *psych* (44), *naniar* (45), and *summarytools* (46) packages. Students with no collected data (*n* = 6; non-participation in PE because of injury) were then removed from the dataset. Little's test was used to assess tenability of data missing completely at random (MCAR; 47). Results of *χ*^2^(766) = 1,681, *p* < .001 (with missing patterns = 91) provided evidence against MCAR. The assumption of missing at random (MAR) was plausible based on the results of a sensitivity analysis of missing data grouped by class. Two items (Rhythmic Gymnastics, and Modern Dance) were eliminated prior to further analysis due to low observed frequency (*n* = 1, and 0, respectively).

#### Dimensionality

All IRT models were estimated using Marginal Maximum Likelihood with the expected-maximization algorithm in *mirt* (version 1.34.11; 48), robust to high degrees of missing data (49). A two-stage analysis was performed. First, sequentially more complex models were estimated until there was no improvement in model-data fit, or convergence issues occurred due to over factoring. We fitted a (1) unidimensional partial credit model (1d-PCM), (i) unidimensional graded response model (1d-GRM), and (ii) exploratory multidimensional correlated GRM (2d-GRM and 3d-GRM). Comparison between models used the likelihood-ratio test (LRT; 50) based on the −2LL statistic for each model (significance level of .05) to assess whether adding parameters (i.e., discrimination) and extra dimensions improved the fit of the model. The Akaike Information Criterion (AIC; 51) and sample-adjusted Bayesian information criterion (SABIC; 52) provided additional insights, with lower values indicating better model fit.

Then, after an optimal exploratory solution was attained, its standardized loadings (*oblimin* rotated) were assessed to identify non-salient items with a threshold of *λ* < .30 (53) or communality <.40. Cross-loadings were assessed using a variance explained ratio (*λ*_1_^2^/*λ*_2_^2^), with values lower than 1.5 (54) considered for elimination depending on factor interpretability. These items were then removed one by one (with model re-estimation) until simple structure was achieved. For the second stage, all previous models were rerun to detect whether the sequential improvement in fit held after removal of items. Finally, item loadings were constrained to load on its salient factor, and a confirmatory GRM model was fit.

In this final solution, the magnitude of standardized loadings and discrimination (slope) parameters were assessed: (a) loadings were interpreted as excellent, very good, good, fair, or poor when higher than .71, .63, .55, .45, and .32, respectively (55); (b) discriminations were interpreted as very high, high, moderate, low, and very low when higher than 1.70, 1.35, 0.65, 0.35 and 0.01, respectively (56).

#### Differential item functioning (DIF)

Before DIF analysis, five cases had to be removed to equalize categories in the Throws and Jumps (both from Athletics) activities. DIF analysis was performed between sexes using a two-stage approach. First, a multiple-group IRT version of the final model was fit with no equality constraints across-groups and used as a reference to run the DIF function in *mirt*—which adds, and tests *via* LRT, equality constraints for one item at a time, returning multiplicity-controlled (57) *p*-values. Three items with the highest *p*-values were selected as anchors (i.e., assumed invariant) and a final addictive sequential analysis was run in the anchored model (i.e., three invariant items constrained to equality), with freely estimated means and variances. Adjusted *p*-values <.05 were used as the threshold for existence of DIF.

#### Discriminant and convergent validity

Bivariate Pearson and polyserial correlations (and 95% CI) were calculated using the *polycor* (58) and *piercer* (59) packages using all pairwise complete observations. These were used to evaluate discriminant validity (threshold of *r* = .85 to discern whether resulting variables were statistically different) and convergent validity based on magnitude reported in similar studies. Magnitudes were interpreted as: very high, high, moderate, and low correlations, when *r* > .90, >.70, >.50, >.30, respectively (60). Inter-factor discriminant validity was assessed *via* correlation in the final MCRT model, using the same .85 threshold.

#### Reliability and scoring

Marginal reliability (61), using Expected a-posterior (EAP) (62) scores, was calculated to quantify average reliability across the *θ* continuum. These were evaluated as acceptable (*ρ_xx_*_ _> .70; 63), and as good (*ρ_xx_*_ _> .80; 64). Thresholds for each item (*d_k_*, or intercept parameter) were transformed into *difficulty* parameters (*b_k_*) using *b_k _= *−(*d_k_*/*a_k_*) (65) for easier interpretation.

## Results

Given the initial focus on the development of the PPLA-O, this section will first describe the results of domain identification and measure selection—including relevant definitions, and a summary literature review of its theoretical framework and relationships with PA participation or other relevant outcomes. It will then present the results of the remaining studies: content analysis, pilot testing, and IRT analysis of the MCRT module.

### Domain identification and measure selection

#### Health-related fitness (HRF) module

Physical fitness can be interpreted as the capacity to perform PA and/or physical exercise that integrates most bodily functions involved in movement (66, 67). Some authors suggest it as a predictor of PA in youth (6, 68), with active youth presenting healthier physical fitness profiles (69). However, this is disputed by other authors (66, 70).

More robust evidence, however, correlates fitness with various health outcomes throughout the life span (71). Among these, cardiovascular endurance is linked with diverse metabolic markers (72), mental health (73, 74), and cognitive benefits including academic performance (75, 76). Musculoskeletal fitness is liked with increased bone density (72) and positive self-perceptions (77). And, despite there being no compelling link between flexibility and health, the former is suggested to be central to correct posture and increased functional capacity (78).

Given its prominent role in a healthy and active life, HRF is an integral part of the PPES, as one of its three major areas, along with *physical activities* and *knowledge*. Its assessment is operationalized through the FITescola© test battery (21). This battery, analogous to FitnessGram© (78), offers a set of protocols to assess whether children and adolescents meet evidence-based criteria for health-related benefits. From these, we selected the most disseminated ones in PE teacher's practice, that simultaneously adhere to international recommendations (72, 79) (Table 2, column 5), and have extensive validity and reliability evidence (80–85). The obtention of the Healthy Fitness Zone was mapped as the transition point between *Foundation* and Mastery level for elements in this module, with the Athletic Profile values used as a reference for maximum points. The latter is a zone designed to assess athletic potential in youth (86).

**Table 2:** Domain identification for the physical and cognitive domain of the PPLA-observation instrument (PPLA-O).

	Theoretical framework	Operational definition	Definition per level	Instruments/Measures	PPLA-O Module
**Physical Domain**					
**Health-related Fitness**					
Cardiorespiratory Endurance	FITescola©	The ability of the heart and lungs to deliver oxygen to working muscle	Foundation: Building health-related fitness that allows for a functional lifestyle and health-related benefits Mastery: Building health-related physical fitness necessary for excelling in performance-driven settings	PACER/20-meter shuttle run^b^	Health-Related Fitness (HRF)
Muscular Endurance		The ability of muscle(s) to repeatedly exert force over a sustained period		Curl-ups (core endurance)^b^ 90° push-ups (upper-body endurance)^b^	
Flexibility		The capacity of a joint or muscle to move through its full range of motion		Backsaver Sit-and-reach (lower flexibility)^b^ Shoulder Stretch (upper flexibility)^b^	
Movement Competence					
Locomotion	(27, 117)	Movement skills that allow a person to move from one place to another (in multiple environments)^a^	Foundation: application of baseline skills and techniques in reduced settings (exercises, reduced or constrained gameplay) (Introductory level in the PPES) Mastery: application in settings representing the physical activity (global, formal level of participation) (Elementary level in the PPES)	Teacher-reported proficiency levels in Physical Activities in PE	Movement Competence, Rules, and Tactics (MCRT)
Object Manipulation		Movement skills that use a body part to move or manipulate an object			
Stability/Balance		Skills involving balance and weight transfer^a^			
Moving with equipment	(9)	Movement skills used to move on, in, or with, equipment from one place to another			
**Cognitive Domain**					
Rules	(2, 9)	Explicit or understood regulations and principles governing conduct or procedure with movement and PA	Foundation: Knowledge and compliance with safety rules and regulations of activities Mastery: Active participation in the enforcement or adaptation of rules	Teacher-reported proficiency levels in Physical Activities in PE	
Tactics	(8, 9, 30)	Planed and *ad hoc* decisions and actions, employed in the moment for the pursuit of goals	Foundation: Accumulation and application of simple tactics to solve a problem (single constraints) Mastery: Relational application of tactics in response to multiple constraints		

PA, physical activity; PACER, progressive aerobic cardiovascular endurance run; PE, physical education; PPES, portuguese PE syllabus.

^a^ According to the Australian Physical Literacy Framework (2).

^b^ FITescola® (21).

#### Movement competence, rules, and tactics (MCRT) module

##### Movement competence

Movement competence (MC) can be defined as the development of sufficient movement skills to assure successful performance in a variety of physical activities, be that work or play (26, 87). This concept is employed by Whitehead (88) in allusion to a “bank” that enables individuals to respond automatically and meaningfully to movement situations. Most commonly, these skills are divided into (1) fundamental movement skills, and (2) specialized movement skills (27). Fundamental movement skills are organized series of basic movements that involve combinations of two or more body segments (27), and form the building block for specialized movement skills (89), which represent application of these fundamental movement skills to specific physical activity or sports contexts with increased refinement (e.g., fielding a ground ball; 27, 28). Different, yet analogous taxonomies include the subdivision into general, refined, and specific movement patterns (90). All these movement skills can be categorized into different movement skill sets according to their function (26) as *locomotor*, *stability,* or *manipulative* movement skills (27), and present multiple phases and stages of development throughout the lifespan. Other sources add a fourth category that includes movement skills with equipment (e.g., bike, surfboard, skate rollers; 2, 9).

MC has a suspected cause-effect relationship with PA (91), with multiple reviews identifying a positive association between the two across childhood (92). This association also seems to be higher with object control/manipulative movement skills (93, 94).

However, few studies have examined this correlation among adolescents (92). Similarly, positive correlations have been identified with perceived competence (95) and health-related fitness (5, 96).

In the PPES, MC is developed within the physical activities area, which includes subareas for diverse physical activities (i.e., Team sports, Gymnastics, Athletics, Racquets, Combat, Rollerskating, Swimming, Rhythmic-Expressive, Traditional Games, and Nature exploration). In each of these subareas, multiple physical activities (to which we will refer simply as *activities*, from now on) are used as a means of development and assessment of each student through three levels: Introductory, Elementary, and Advanced. The Introductory level frames multiple foundational skills and knowledge needed for participation in each activity—in reduced or constrained gameplay, or pedagogical progressions leading to the formal setting of the activity. The Elementary level refers to the mastery of the main elements of each activity—in the full formal setting of the activity. The Advanced level establishes skills and knowledge needed for higher-degree participation in the activities (e.g., performance-settings). This assessment uses a set of rubrics that establish (1) the skill, knowledge, or attitude to be observed, (2) the context (e.g., 2 × 2 reduced gameplay of volleyball, or a gymnastics sequence composed of predetermined movements, and c) multiple qualitative criteria that describe the action. Given the above frame, we corresponded to the Introductory and Elementary levels in these activities with the *Foundation* and *Mastery* levels of the PPLA in all elements of movement competence (i.e., locomotion, manipulative, stability, moving with equipment).

##### Rules

Although framed within the realm of team sports and games, most literature on rules readily generalizes to other movement contexts. Rules provide a structure that manages and guides practitioners' actions (97). These can be considered primary, or fundamental, when they act as constraints that regulate and apply restrictions on the mode of action available to the individual (e.g., scoring rules); or as secondary when they represent written or unwritten rules that facilitate participation [e.g., safety and ethical rules of organized PA; (9)]. Both contribute to the *form* of the activity as we know it (16). Understanding rules and their application is therefore an essential part of every activity—something that Bunker and Thorpe frame as “Game Appreciation” (8).

Within the PPES, rules' knowledge and understanding are integrated holistically within each activity proficiency level previously mentioned. Thus, all activities promote the learning of safety codes and equipment management, while activities like Team Sports and Athletics allow learning of more closed scoring and playing rules. These outcomes are framed into the *Foundation* level of this element. At higher levels (mostly Advanced), students are asked to be officials and referees, which works as a powerful learning tool to reinforce rule knowledge and conditional application of all aspects of the activity (16). This skill is proposed as part of the *Mastery* level.

##### Tactics

Tactics can be framed as time-sensitive responses to problems posed in movement and PA contexts, be that inherent to game participation (i.e., acquiring advantage), or informal PA (i.e., maximizing quality and efficiency) (9, 98). These contexts act as eventful *dynamic systems* (99) that require participants to develop and apply higher-level cognitive skills (e.g., comparing, contrasting, analyzing, evaluating) required for thoughtful decision-making (100), in interaction with others and the environment (9). Despite being separated here into two different elements, tactical knowledge and application are mostly conceived as the next (higher-order) level of rules' knowledge, in a learning continuum that frames decision-making within PA (8, 9, 97): Only after participants can identify the constraints imposed by rules, can they acknowledge degrees of freedom available to act.

*Game sense* approaches, which propose teaching of PA through reduced or adapted forms of the formal activity [e.g., Teaching Games for Understanding (TGfU); 8], recognize that the learning of specific skills and tactics constrains each other (101); while *traditional*, skill-centered approaches (i.e., analytical) focus on the former as the main constrainer of the capacity to participate in PA. The TGfU approach recognizes the similarity between tactical actions among the various games by categorizing them into (1) target games, (2) net/wall games, (3) striking/fielding, and (4) invasion games (8). Based on this taxonomy, the *Game Performance Assessment Instrument* typifies tactical action these into six transversal categories: (1) decision-making, (2) adjust, (3) cover, (4) support, (5) guard/mark, (6) base (30, 102)—skill execution excluded.

Benefits of using these approaches might include increased engagement, enjoyment, and motivation in PE classes (103). Also, some authors argue that awareness and decision-making skills might transfer to contexts outside of movement (2, 9), being central to critical thinking as a general education outcome (100).

As aforementioned, the PPES frames tactical skills within the learning of activities and into the diverse levels of learning. Assessment is made in-context, through a combination of skills and decision-making, coherent with principles of *authentic assessment* (16, 104). We framed a more constrained application of tactics (i.e., reproduction of descriptive tactics) to the *Foundation* level, while a more critical, relational stance on decision-making was framed at the *Mastery* level.

Given the integrated nature of the *Movement Competence*, *Rules*, and *Tactics* elements, the specification levels for each activity were selected as holistic, process-oriented measures of these elements. A set of 22 physical activities that represent the full breadth of subareas within the syllabus were chosen, with the possibility for teachers to include any other activity assessed. Chosen activities spanned all movement forms (90, 105) and two of the four game types according to TGfU (Table 3). Target and striking games are not commonly developed in Portuguese PE and were not included.

**Table 3:** Content analysis of the Portuguese physical education (PE) syllabus.

Physical Activity	Classification			Content analysis																		
	Movement Form (90, 105)	Portuguese PE Syllabus (12–15)	TGfU/GCS (8, 118)	Locomotion Skills^a^ max. points 8			Manipulative Skills^a^ max. points 13			Stability/Balance Skills^a^ max. points 10			Moving with equipament^b^ max. points 6			Tactics^c^ max. points 5			Rules^d^			
				I	E	A	I	E	A	I	E	A	I	E	A	I	E	A	Safety rules	Specific rules	Referee signals	Participations as referee/judge
Races (Athletics)	Athletic	Athletics		2	2	2				2	3	3							I			A
Throws (Athletics)	Athletic	Athletics			1	2	1	1	1	1	4	4							I			A
Jumps (Athletics)	Athletic	Athletics		2	3	3				4	5	5							I			A
Wrestling	Competitve	Combat		1	1	1		1	1	5	5	5				2	3	3	I	I	E	E
Judo	Competitve	Combat		1	1	1	1	1	1	6	6	6				2	3	3	I	I	I	
Floor Gymnastics	Athletic	Gymnastics			2	2				5	8	8							E			
Artistic Gymnastics	Athletic	Gymnastics		2	2	2				4	6	8							I			
Acrobatic Gymnastics	Athletic	Gymnastics		3	3	3				7	8	8							I	I		
Rhythmic Gymnastics	Aesthetic and Expressive	Gymnastics		2	3	3	3	4	4	3	8	8							I			A
Handball	Competitive	Team Sports	Invasion	3	3	4	3	3	3	3	5	5				2	5	5	I	I	E	A
Football	Competitive	Team Sports	Invasion	2	3	3	5	5	5	3	5	5				3	3	4	I	I	E	A
Basketball	Competitive	Team Sports	Invasion	4	4	4	4	4	4	4	6	6				2	4	4	I	I	E	A
Rugby	Competitive	Team Sports	Invasion	2	3	3	4	5	5	4	5	5				3	5	5	I	I	E	
Orienteering	Adventure	Nature Exploration		1	1	1				2	2	2				1	1	1	I	I		
Climbing	Adventure	Nature Exploration		1	1	1				3	4	4				1	1	1	I	E		
Rollerskating^e^	Athletic/Aesthetic and Expressive	Rollerskating			2	2				4	6	6	1	1	1				I			A
Table Tennis	Competitive	Racquets	Net							3	3	3				1	1	4		I		A
Badminton	Competitive	Racquets	Net	1	2	2				5	6	6				2	2	4		I		A
Volleyball	Competitive	Team Sports	Net	2	3	3				4	5	6					3	4	I	I	E	
Dance (Modern)	Aesthetic and Expressive	Rhytmic and Expressive		6	7	7				4	5	5										A
Dance (Social)	Interpersonal/Relational	Rhytmic and Expressive		1	1	3				3	3	3							I			
Aerobics	Fitness & Health	Rhytmic and Expressive		3	6	6				2	6	6										

TGfU, teaching games for understanding; GS, game sense; I, introductory proficiency level; E, elementary proficiency level; A, advanced proficiency level.

^a^ Based on (27).

^b^ Based on (9).

^c^ Based on Game Performance Assessment Instrument items (30), extended to general decision-making in all activities.

^d^ Level at which item appears.

^e^ After Introductory level, Rollerskating takes the form of (i) Rollerskates Racing, (ii) Artistic Rollerskating, or (iii) Hockey in Rollerskates – analysis presented here refers to (ii).

### Content analysis

Table 3 presents the summary of the content analysis of the PPES. Higher levels of proficiency in each activity entailed a higher diversity of movement skills in all typologies; however, this tendency only emerged between the Introductory and Elementary levels, with almost no new movement skills required when transitioning to the Advanced level. Locomotor skills were required with similar diversity across all types of activities, with two clusters emerging according to manipulative skills (mostly Team Sports) and stability (Gymnastics and Rollerskating) movement skills: while Team Sports required mostly dynamic balancing, twisting, turning, landing, and dodging movement skills, Gymnastics uniquely required skills combining inverted support, rolling, and diverse bending and stretching movement skills. Tactics-wise, a similar pattern was noted with increasing levels requiring a higher diversity of tactical action—without the plateau observed for movement skills. As expected, tactical actions were mostly requested by Team Sports and Racquets activities.

Finally, regarding rules, four general categories emerged from the analysis. Knowledge and application of safety rules and specific activity rules were mostly observed at the Introductory levels; while identification of referee signals, and officiating were mostly skills required for Elementary and Advanced levels, respectively.

### Pilot testing

Teachers had no difficulties with data insertion and regarded the instructions as clear. As expected, data collection implied no further efforts, as activities and HRF protocols were already part of their lessons. They highlighted errors in the code generator spreadsheet and PPLA-O spreadsheet, which were corrected for the next phase.

#### Preliminary analysis

Seven activities had lower than 90% assessment rate (Modern Dance, Rhythmic Gymnastics, Rugby, Wrestling, Judo, Acrobatic Gymnastics, and Tennis; Table 4). The most prevalent level of proficiency was Introductory, with the Advanced level attaining only residual prevalence (0 to 5.1% of assessed students). Flexibility protocols had lower percentages of assessed students compared to other protocols (Table 5).

**Table 4:** Descriptive statistics for teacher-reported proficiency levels in physical activities – movement competence, rules, and tactics module (*N* = 515).

Physical Activity	Missing cases (%)	Observed Proficiency Levels			
		Non-Introductory^a^	Introductory	Elementary	Advanced
Races (Athletics)	187 (36.3%)	22 (6.7%)	180 (54.9%)	126 (38.4%)	
Throws (Athletics)	346 (67.2%)	2 (1.2%)	87 (51.5%)	80 (47.3%)	
Jumps (Athletics)	392 (76.1%)	5 (4.1%)	87 (70.7%)	31 (25.2%)	
Wrestling	491 (95.3%)	10 (41.7%)	14 (58.3%)		
Judo	490 (95.1%)	3 (12%)	22 (88%)		
Floor Gymnastics	32 (6.2%)	91 (18.8%)	320 (66.3%)	72 (14.9%)	
Artistic Gymnastics	53 (10.3%)	85 (18.4%)	271 (58.7%)	104 (22.5%)	2 (0.4%)
Acrobatic Gymnastics	475 (92.2%)	14 (35%)	26 (65%)		
Rhythmic Gymnastics	514 (99.8%)	1 (100%)			
Handball	114 (22.1%)	78 (19.5%)	212 (52.9%)	111 (27.7%)	
Football	64 (12.4%)	116 (25.7%)	179 (39.7%)	133 (29.5%)	23 (5.1%)
Basketball	43 (8.3%)	84 (17.8%)	265 (56.1%)	123 (26.1%)	
Rugby	500 (97.1%)	8 (53.3%)	7 (46.7%)		
Orienteering	345 (67%)	1 (0.6%)	82 (48.2%)	87 (51.2%)	
Climbing	410 (79.6%)	11 (10.5%)	61 (58.1%)	33 (31.4%)	
Rollerskating	338 (65.6%)	84 (47.5%)	76 (42.9%)	17 (9.6%)	
Table Tennis	297 (57.7%)	23 (10.6%)	141 (64.7%)	54 (24.8%)	
Badminton	8 (1.6%)	56 (11%)	264 (52.1%)	163 (32.1%)	24 (4.7%)
Volleyball	5 (1%)	40 (7.8%)	295 (57.8%)	163 (32%)	12 (2.4%)
Dance (Modern)	515 (100%)				
Dance (Social)	204 (39.6%)	53 (17%)	208 (66.9%)	48 (15.4%)	2 (0.6%)
Aerobics	395 (76.7%)	4 (3.3%)	96 (80%)	20 (16.7%)	
Tennis	469 (91.1%)	2 (4.3%)	38 (82.6%)	6 (13%)	

^a^ Non-introductory level refers to students that have yet to achieve the standards for the Introductory level.

**Table 5:** Descriptive statistics for teacher-reported results for the health-related fitness module (*N* = 515) and their reference thresholds.

Health-Related Fitness Measures	Missing cases (%)	M (SD)	Median	Healthy Fitness Zone threshold (Female/Male)^a^	Athletic Profile threshold (Female/Male)^a^
PACER (laps)	22 (4.2%)	49.5 (22)	44.0	29/42	48/85
Push-ups (executions)	26 (5.0%)	18.1 (9.6)	18.0	18/24	62/71
Curl-ups (executions)	23 (4.4%)	48.6 (21.7)	45.0	7/16	17/27
**Shoulder Stretch (% of achievement)**					
Right	83 (15.9%)	95%			
Left	83 (15.9%)	89%			
**Sit-and-Reach (cm)**					
Right	85 (16.3%)	30.7 (8.3)	31.0	30.5/20.3	35.3/33.5
Left	84 (16.1%)	30.2 (8.2)	31.0		

^a^ For a 15-year-old adolescent, according to the FITescola® website.

### IRT analysis of the movement competence, rules, and tactics module

#### Dimensionality

In the first stage of analysis, the 2d-GRM presented the best fit according to information criteria (AIC, SABIC, and −2LL; Table 6). According to the likelihood-ratio test (LRT), freely estimating discrimination (slope) parameters improved the fit from the 1d-PCM to the 1d-GRM; and estimating an additional dimension also improved fit from the 1d-GRM to the 2d-GRM. A 3d-GRM was estimated, however, its information matrix could not be inverted, signaling an empirically unidentified model (estimates are not presented).

**Table 6:** Model fit indices and statistics for the movement competence, rules, and tactics module.

	AIC	SABIC	−2LL	LRT	Removed items (reasons)
**First stage**					Aerobics, Tennis, Social Dance (non-salient loadings) Orienteering (low communalities) Judo, Rugby (SE larger than slope parameters) Acrobatic Gymnastic, Wrestling (problematic cross-loadings)
1d-PCM	8,360.60	8,407.67	8,272.59		
1d-GRM	8,026.04	8,094.51	7,898.03	Δ*χ*^2^(20) = 374.56*, p* < .001	
2d-GRM (E)	7,889.53	7,979.41	7,721.53	Δχ^2^(20) = 176.50, *p *< .001	
**Second stage**					
1d-PCM	7,112.58	7,145.75	7,050.58		
1d-GRM	6,928.36	6,974.37	6,842.36	Δχ^2^(12) = 208.22, *p* < .001	
2d-GRM (E)	6,788.18	6,847.03	6,678.18	Δχ^2^(12) = 164.18, *p *< .001	
2d-GRM (C)	6,861.48	6,908.55	6,476.16	Δχ^2^(11) = 95.29, *p *< .001^a^	

1d, unidimensional, 2d, multidimensional model with 2 correlated factors; (E), exploratory; (C), confirmatory; AIC, Akaike's information criteria; SABIC, sample-adjusted Bayesian information criteria; −2LL, −2* Log-Likelihood; LRT, Likelihood ratio test.

^a^ In favor of the exploratory model.

Item standardized loadings and parameters were analyzed based on the 2d-GRM exploratory solution. Reasons for item removal are presented in Table 6. As a note, Wrestling item had a borderline variance ratio (1.66), and we opted initially for non-removal based on its added value as a unique item concerning Combat activities. However, estimation of the following second stage confirmatory 2d-GRM (with items constrained to load on its salient factor) did not converge. Removal of this item allowed the solution to converge.

The second stage comprised sequential re-estimation of all models, without removed items, to assess whether results obtained in the first stage were robust. Improvement in fit between models was equivalent to those observed during the first stage. Finally, a confirmatory 2d-GRM was fit, resulting in decreased fit (according to all indices) vs. its exploratory counterpart, which was expected since the former imposes more constraints on item loadings (cross-loadings constrained to 0).

**Figure 1:**
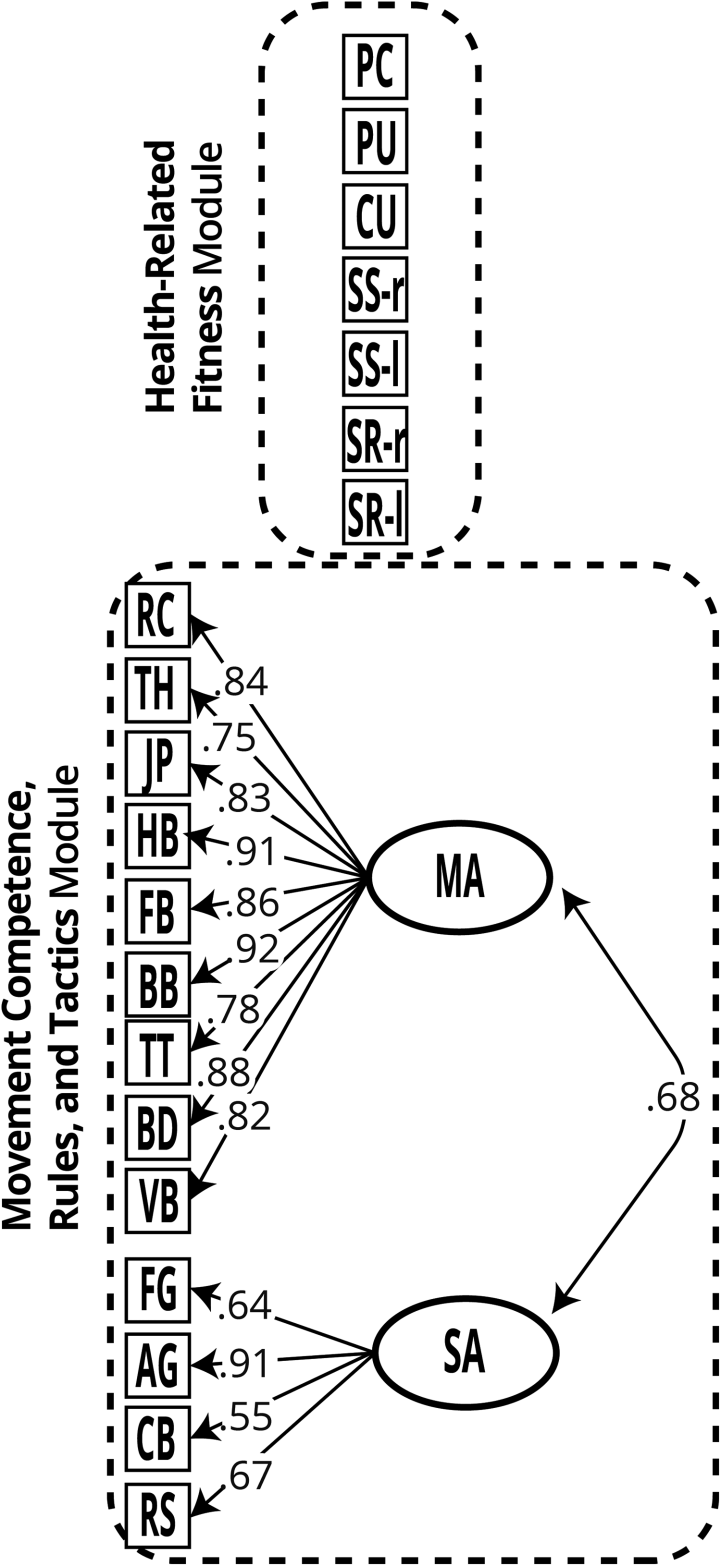
Portuguese Physical Literacy Assessment - Observation (PPLA-O) two modules, with estimated parameters for the movement competence, rules, and tactics module (2-dimensional graded response model). Legend: PC, Pacer; PU, Push-ups; CU, Curl-ups; SS-r, Shoulder Stretch (right); SS-l, Shoulder Stretch (left); SR-r, Backsaver Sit and Reach (right); SR-l, Backsaver Sit and Reach (left); RC, Races (athletics); TH, Throws (athletics); JP, Jumps (athletics); HB, Handball; FB, Football; BB, Basketball; TT, Table Tennis; BD, Badminton; VB, Volleyball; FG, Floor Gymnastics; AG, Artistic Gymnastics; CB, Climbing; RS, Rollerskating; MA, Manipulative-based Activities; SA, Stability-based Activities.

Loadings in the final confirmatory solution ranged from very good to excellent (.75 to .92, and .64 to .91), for dimensions 1 and 2, respectively (Table 7, Figure 1). An equivalent pattern of moderate (*a* > .65) to very good (*a* > 1.70) discrimination parameters (56) indicates that items are performing correctly in their respective dimension (i.e., providing information to separate students with different levels of *θ*). Interpretation of these two moderately (*r* = .68) correlated dimensions is coherent with items (i.e., PA) being better measures of either Manipulative skills, or Stability skills, as such we named these dimensions as *Manipulative-based Activities* (MA), and *Stability-based Activities* (SA), respectively (Table 7). Usage of Locomotion skills is likely prevalent across all activities, and thus no third factor emerged based on it. Surprisingly, all Athletics disciplines had higher loadings on the Manipulative factor than on the Stability factor; also, loadings patterns do not suggest that tactical skills might be a source of covariation among tactical-alike activities (e.g., Handball and Basketball). Interpretations for these occurrences are provided in the Discussion.

**Table 7:** Item parameters, inter-factor correlations and reliability for 2-dimensional graded response model.

	Exploratory			Confirmatory							
				Standardized loadings			Slope parameters		Intercept parameters		
	Manipulative–based Activities	Stability-based Activities	Communalities	Manipulative–based Activities	Stability-based Activities	Communalities	a_1_ (SE)	a_2_ (SE)	d_1_ (SE)	d_2_ (SE)	d_3_ (SE)
Races (Athletics)	**.** **66**	.34	.74	.84		.71	2.66 (.33)		4.47 (.46)	−1.24 (.23)	
Throws (Athletics)	**.** **87**	−.18	.66	.75		.57	1.95 (.42)		4.34 (.76)	−1.16 (.30)	
Jumps (Athletics)	**70**	.24	69	.83		69	2.53 (.52)		4.41 (.70)	−2.46 (.46)	
Handball	**.** **74**	.31	.84	.91		.83	3.78 (.45)		2.95 (.37)	−2.89 (.36)	
Football	**.** **81**	.09	.73	.86		.73	2.77 (.25)		2.00 (.23)	−1.44 (.21)	−5.40 (.43)
Basketball	**.** **85**	.14	.84	.92		.84	3.94 (.46)		3.91 (.45)	−2.79 (.35)	
Table Tennis	**.** **95**	−.25	.76	.78		.61	2.12 (.31)		3.77 (.44)	−1.54 (.25)	
Badminton	**.** **94**	−.07	.83	.88		.78	3.20 (.30)		4.51 (.40)	−1.25 (.22)	−5.94 (.50)
Volleyball	**.** **70**	.23	.68	.82		.67	2.41 (.22)		4.27 (.33)	−1.20 (.18)	−5.95 (.47)
Floor Gymnastics	−.10	**.** **83**	.64		.64	.41		1.41 (.17)	1.97 (.18)	−2.29 (.19)	
Artistic Gymnastics	.28	**.** **67**	.62		.91	.83		3.75 (.95)	3.72 (.80)	−3.06 (.67)	−11.09 (2.43)
Climbing	.07	**.** **61**			.55	30		1.12 (.37)	2.53 (.41)	−1.01 (.27)	
Rollerskating	.18	**.** **64**	.48		.67	.45		1.53 (.32)	.76 (.25)	−2.48 (.33)	
											
Marginal Reliability	.88	.67		.89	.73						
Correlation	.43		.68								

SE, standard error.

Note: salient loadings in each factor are bolded in the exploratory model.

#### Differential item functioning (DIF)

In the first stage of the analysis, the Throws (Athletics), Climbing, and Rollerskating indicators were selected as anchors (adjusted *p*-values = 1.00). Subsequent sequential analysis with these indicators constrained to equality across-groups revealed no DIF according to sex.

#### Discriminant and convergent validity

Inter-factor correlation between MA and SA was moderate to high (*r* = .68; Table 7). Table 9 displays the bivariate correlations between all variables in both PPLA-O modules, along with an additional BMI variable. These results will be discussed and compared further in the Discussion.

**Table 8:** Difficulty of each physical activity proficiency level transition point (threshold).

	*b* (difficulty)		
	Non-Introductory to Introductory	Introductory to Elementary	Elementary to Advanced
**Manipulative–based Activities**			
Races (Athletics)	−1.68	0.47	
Throws (Athletics)	−2.23	0.59	
Jumps (Athletics)	−1.74	0.97	
Handball	−0.78	0.76	
Football	−0.72	0.52	1.95
Basketball	−0.99	0.71	
Table Tennis	−1.78	0.73	
Badminton	−1.41	0.39	1.86
Volleyball	−1.77	0.50	2.47
**Median**	**−1** **.** **68**	**0** **.** **59**	**1** **.** **95**
**Stability-based Activities**			
Floor Gymnastics	−1.40	1.62	
Artistic Gymnastics	−0.99	0.82	2.96
Climbing	−2.26	0.90	
Rollerskating	−0.50	1.62	
**Median**	**−1** **.** **19**	**1** **.** **26**	**2** **.** **96**

**Table 9:** Pearson and polyserial bivariate correlation matrix for PPLA-O variables.

	1.	2.	3.	4.	5.	6.	7.	8.	9.	10.
1. Age										
2. MA	.23 [.15, .31]									
3. SA	.18 [.09, .26]	.79 [.75, .82]								
4. BMI	.05 [−.05, .14]	−.04 [−.13, .06]	−.13 [−.22, −.03]							
5. PACER	−.06 [−.14, .03]	.37 [.29, .44]	.31 [.23, .39]	−.25 [−.34, −.16]						
6. 90° Push-ups	.03 [−.05, .12]	.43 [.35, .50]	.35 [.27, .43]	−.18 [−.27, −.09]	.61 [.55, .66]					
7. Curl-ups	−.04 [−.13, .05]	.34 [.26, .42]	.27 [.19, .35]	−.19 [−.28, −.10]	.44 [.37, .51]	.41 [.33, .48]				
8. Shoulder Stretch (Right)^a^	−.06 [−.20, .09]	−.40 [−.51, −.27]	−.33 [−.45, .20]	−.18 [−.31, −.03]	−.04 [−.19, .11]	.00 [−.15, .15]	.05 [−.10, .19]			
9. Shoulder Stretch (Left)^a^	−.20 [−.31, −.07]	−.36 [−.47, −.25]	−.28 [−.39, −.16]	−.26 [−.37, −.14]	−.03 [−.16, .10]	−.05 [−.18, .08]	−.05 [−.17, .08]	.71 [.62, .78]		
10. Backsaver sit-and-reach (Right)	.00 [−.09, .09]	−.24[−.33, −.15]	−.04 [−.13, .05]	.01 [−.08, .11]	−.14 [−.23, −.05]	−.08 [−.17, .02]	−.05 [−.14, .05]	.28 [.14, .41]	.30 [.17, .41]	
11. Backsaver sit-and-reach (Left)	.00 [−.09, .10]	−.22[−.30, −.13]	−.01 [−.10, .08]	−.01 [−.10, .09]	−.14 [−.23, −.05]	−.05 [−.14, .05]	−.01 [−.11, .08]	.29 [.15, .42]	.29 [.16, .40]	.93 [.92, .95]

MA, manipulative-based Activities; SA, stability-based activities; BMI, body mass index; PACER, progressive aerobic cardiovascular endurance run.

^a^ Polyserial correlations in these rows.

#### Reliability and scoring

Both dimensions of the MCRT attained acceptable marginal reliability in the final solution (*ρ_xx_*_ _= .89 and.73, respectively; Table 7). Table 8 presents transformed intercept parameters (category threshold) which can be interpreted as transition points between levels of proficiency for each activity (i.e., *θ* point at which there is a 50% probability to be scored in that category or higher; 109). Median values represent a heuristic cut-score between general proficiency levels (*θ*) in each dimension. I.e., a student with *θ* = −1.68 is likely transitioning from Non-Introductory to Introductory level in most Manipulative activities.

## Discussion

Our aims for the following studies were to (a) develop the PPLA-Observation based on the review of relevant conceptual frameworks and Portuguese PE syllabus practices; (b) investigate the dimensionality structure of one of its modules—Movement Competence, Rules, and Tactics module—through Item Response Theory (IRT) methods; (c) test this structure for differential item functioning according to sex; (d) establish support for convergent and discriminant validity, and score reliability for this module. A secondary aim was to draw inferences for scoring and criterion-referenced cut-scores mechanisms.

### IRT analysis of the movement competence, rules, and tactics module

#### Dimensionality

Our results, based on exploratory and confirmatory IRT analysis, provide evidence in favor of a two correlated factor solution for assessing Movement Competence, Rules, and Tactics, with evidence of measurement invariance (no-DIF) across sexes. This is contrary to our initial conceptualization that proposed that seven latent variables could be responsible for the variance in observed proficiency levels of activities: Locomotion, Manipulative, Stability, and Movement skills using Objects, Rules, and Tactics. Items (activities) did not cluster according to different tactical typologies, movement forms, or subareas. Instead, our results suggest that their variance is driven according to competence in two types of movement skills: Manipulative movement skills, and Stability movement skills. Competence in Locomotor movement skills did not emerge as a latent factor explaining variance. This might be due to locomotor skills being transversally required in specialized skills in all evaluated activities (e.g., sliding to hit a falling shuttlecock, or running and then jumping onto a trampoline)—as can also be seen in our content analysis of movement skills (Table 3).

Another unexpected finding was that two Athletics disciplines that were expected to load on the SA dimension (i.e., Running, and Jumps)—as specific skills for these activities are mostly locomotor and stability-based—presented higher loadings on MA. This might originate from a disconnect on how this group of activities (Athletics) is conceived and assessed within the PPES: rubrics for all disciplines are grouped and assessed as a single activity, however, throughout the syllabi (12), the three disciplines appear mentioned as different activities. It is possible that this led to teachers reporting according to different standards. This requires scrutiny and caution in further developments of this tool.

Regarding *Tactics*, content analysis of the PPES revealed that until the Elementary proficiency level, both movement skills, and tactical requisites increase simultaneously. It is during the transition to the Advanced level that tactical indicators take precedence (Table 3). It is plausible that skill and tactical factors co-vary closely until the Elementary level, and only when students transition into Advanced levels is the tactical factor singularly driving variance in items—since movement skills factors cease or lower their effect at this level. However, in our sample, almost all students were at, or below, the Elementary level in all activities (Table 4), which could preclude disentanglement of variance between these factors. Also, since most tactical-heavy activities are those requiring manipulative skills, the MA factor might likely be accounting for variance of tactical knowledge and application. Further studies with large-scale samples, with a higher proportion of students in Advanced stages, could test these hypotheses and offer insights into this factorial structure.

Regarding *Rules*, variance caused by differing degrees of rule knowledge and application might be similarly overshadowed by movement skills and tactics: A student might know and apply all rules from an activity, but absence of required skill and tactical factors might prevent him from advancing in proficiency level. Albeit aligned with an authentic assessment perspective, this invalidates measurement of this element using only observed activity levels, and will likely require an external instrument (e.g., scale) to isolate.

#### Differential item functioning (DIF)

Items seem to function similarly for both sexes (i.e., no DIF). Results can be meaningfully compared; despite suggestions in the literature pointing to bias when teachers observe MC (18, 107)—considering girl's competence in PA to be below average compared to boys of the same age.

#### Discriminant and convergent validity

The moderate to high correlation between MA and SA (*r* = .68; Table 7) is similar to results of another movement skill battery, using the same conceptualization, in older children and adolescents in a Portuguese sample (*r* = .64 108);. Due to the strength of this correlation, a general motor ability underlying results in both factors is tenable (26), and could be further investigated through second-order or bifactorial modeling (109, 110). Despite this, discriminant validity is still ensured, with inter-factor correlations below .85 (109).

Correlations observed in our study among MA and SA, and correlates like sex, age, BMI, and fitness (Table 9) were coherent with those found in the literature regarding movement skills in adolescents, strengthening the evidence for construct validity of the MCRT. Boys had higher scores than girls in both dimensions (Table 10), with the difference being smaller in stability skills (111, 112). Values for the correlation of age and scores on both dimensions (*r* = .23 [.15, .31], and *r* = .18 [.09, 26], MA and SA, respectively) were like those reported in a meta-analysis by Barnett and colleagues (93)—including an inverse correlation between BMI and SA scores [*r* = −.13 (−.22, −.03)]. Cardiovascular and muscular endurance were also correlated with both scores, in similar magnitude as in previous studies (92, 111). Finally, despite inconclusive results in reviews (92, 96), we observed a negative correlation between all flexibility indicators and scores in both dimensions; this correlation was lower regarding SA, which is plausible with the idea that stability-based activities require higher ranges of motions. The role of flexibility warrants further scrutiny, since our results pointed to a mostly negative correlation with other fitness indicators; especially the sit-and-reach indicators might be collapsed since their correlation suggested they are statistically equivalent (*r* > .85).

**Table 10:** Movement competence, rules, and tactics mean scores stratified by sex for manipulative-based activities (MA) and stability-based activities (SA).

	*θ* (SD)			Transformed scores (SD)		
	Female	Male	Total	Female	Male	Total
MA	−0.30 (0.91)	0.41 (0.84)	0 (0.95)	48.1 (20.9)	64.4 (19.4)	54.9 (21.8)
SA	−0.15 (0.85)	0.21 (0.81)	0 (0.86)	40.6 (16.3)	47.5 (15.5)	43.4 (16.4)

#### Reliability and scoring

Use of a sub-score for each of the identified dimensions of the MCRT seems plausible given the evidence of sub-score reliability. We suggest a transformation so that these scores provide an intuitive 0 to 100 interpretation—like other scores in PPLA. For this transformation, the median *θ* score estimated for the transition from Elementary to Advanced level (*θ* = 1.95, and 2.96, respectively; Table 8) can be used as the upper bound, and the estimated *θ* score for a student with the lowest possible levels in all activities as a lower bound (*θ*_MA_ = −2.38, and *θ*_SA_ = −2.27, not shown). As an example,

$$X_{\mathrm{MA}}\;=\;\frac{\theta +2.38}{\left(1.95+2.38\right)}\times 100$$

with × being the new 0–100 score, and *θ* the estimated *θ*_MA_ score.

Since these scores require complex computations, the effectiveness, and precision of simpler options (e.g., sum-scores) should be investigated in the future, given our concern for feasibility.

Reliability has been widely established for the HRF module protocols. We suggest that results from each protocol should be similarly transformed using the values reported by FITescola® Athletic Profile, based on sex and age, as the upper bound. In this manner, a 0 to 100 criterion-referenced score can be obtained.

### Strengths and limitations

One of the major strengths of the PPLA-O is its feasibility: it uses data routinely collected by PE teachers to frame the evaluated elements into a common reference frame of Physical Literacy. Its content validity is also maximized by making use of (1) HRF protocols that have been chosen and adapted with the PE context in mind (FITescola®), and (2) data referent to proficiency levels in diverse physical activities that were chosen to figure in the Portuguese syllabus by curriculum design experts. It also evaluates movement skills—and inherent tactical actions—within tasks and environmental constraints that will be common to activities practiced outside of PE, providing a chance for an authentic, ecologically valid, and highly feasible assessment. Further efforts could study content and face validity with students and other educational stakeholders, as well as with motor development specialists to provide another layer of validity evidence.

Another strength rests in using IRT methodologies to analyze construct validity and reliability. Due to the intended ecological approach, missing data will always assume large proportions, since different students' needs will dictate that each class will work on and assess different activities. IRT algorithms were specifically designed to work with categorical data and are robust to missing data, using all information available to estimate parameters that also have higher degrees of invariance from sample to sample (53, 113). As such, students with just a few assessed activities will still be able to be scored. However, large amounts of missing data still posed a limitation regarding assessment of absolute fit of the models—through statistical tests equivalent to chi-square (i.e., C2; 113) and derived relative fit indexes (root mean square error of approximation).

One limitation of this study lies in the unknown inter and intra-observer reliability of PE teachers while assessing both the fitness protocols and activity levels. We would argue that numerous factors could contribute to higher reliability, including (1) extensive training during initial teacher's education, (2) clear and task-specific rubrics for each activity and level available in the syllabus (115), (3) specific fitness protocols with detailed instructions and resource for application, (4) collaborative training and observation opportunities within schools, and (5) assessment based on multiple in-context observations. Despite this, these inferences require further scrutiny and empirical validation, since process-oriented assessments are more susceptible to bias caused by different levels of observer's expertise (e.g., 115, 116). As part of this effort, demographic data on PE teachers, along with teaching experience and other relevant variables should also be collected to better understand assessment patterns, which we did not do during these studies.

A final, more general limitation is concerned with the timeframe of this study. All data collection was done amongst lockdowns imposed by the COVID-19 pandemic. This limited the number and quality of activities assessed by PE teachers (especially those involving physical contact like wrestling or acrobatic gymnastics) and might have imposed additional unforeseen limitations on these results. As such, these results should be replicated in a larger, more representative sample of students in regular PE circumstances, which will likely enable a deeper insight into the *Tactics* element.

## Conclusion

Throughout this article, we detailed the development of the PPLA-O, an instrument that assesses the physical and part of the cognitive domains of PL in grade 10 to 12 adolescents (15–18 years). It is composed of two modules, (1) Health-Related Fitness (HRF), and (2) Movement Competence, Rules, and Tactics (MCRT), that integrate observational data from PE teachers into a common frame of criterion-referenced PL (Figure 1). The former makes use of data collected through widely validated FITescola® assessment protocols, while the latter makes use of teacher-reported data collected in a wide range of activities and movement pursuits to measure movement competence and inherent cognitive skills (*Tactics* and *Rules*). We also gathered initial evidence supporting construct validity and score reliability of the MCRT module through IRT multidimensional models. A final two-dimensional graded response model solution (Manipulative-based Activities, and Stability-based Activities) showed best fit to the data. The absence of Differential Item Functioning allows meaningful comparison of scores between sexes. Further studies should assess inter and intra-rater reliability and criterion-related validity. This highly feasible instrument can be used routinely—alongside the other instrument of PPLA (PPLA-Q)—to provide students with feedback on their PL journey and support pedagogical decisions at multiple levels (e.g., class, school, municipality, country).

## Data Availability

The datasets presented in this article are not readily available because participants of this study did not explicitly agree for their data to be shared publicly. Requests to access the datasets should be directed to João Mota, joao.mota@ucc.ie.
